# Age Influences the Bacterial Composition of Samples From Buffaloes in the Marajó Archipelago, Pará, Brazilian Amazon

**DOI:** 10.1111/1758-2229.70330

**Published:** 2026-04-28

**Authors:** Allana Lais Alves Lima, Savio Souza Costa, Rosiane do Socorro dos Reis, Damazio Campos de Souza, Guilherme Costa Baião, Rennan Garcias Moreira, Herve Louis Ghislain Rogez, Diego Assis das Graças, Rinaldo Batista Viana, Joana Montezano Marques

**Affiliations:** ^1^ Institute of Health and Animal Production, Federal Rural University of Amazon Belém Brazil; ^2^ Institute of Biological Sciences, Federal University of Pará Belém Brazil; ^3^ Augusto Motta University (UNISUAM) Rio de Janeiro Brazil; ^4^ Institute of Biological Sciences, Federal University of Minas Gerais Belo Horizonte Brazil; ^5^ Center for Natural and Human Sciences, Federal University of ABC São Carlos São Paulo Brazil

**Keywords:** *Bubalus bubalis*, calf buffalo, lactations, *Metabarcoding*, milk

## Abstract

Buffalo milk stands out for its nutritional qualities and is mainly produced in Brazil in the Amazon region, particularly in the Marajó Archipelago, State of Pará. In this context, the milk microbiome is an intriguing and underexplored research topic. This study tested the hypothesis that the buffalo milk microbiome could share taxa with faeces, pasture soil and calf spittle. A possible correlation between generations was also explored. Samples of milk, faeces, spittle and pasture soil were analysed using a 16S rRNA metabarcoding approach, revealing significant phylogenetic and ecological differences among matrices. Beta‐diversity analysis showed clustering between milk samples from heifers and cows, as well as proximity between soil and buffalo samples (milk and faeces). Relative abundance analysis identified shared taxa between buffaloes and their calves, though no clustering was observed between generations. A total of 15 bacterial families were found across all matrices, with *Staphylococcaceae*, *Planococcaceae* and *Enterobacteriaceae* being the most prominent. The milk microbiota was similar among animals of different ages. The grouping pattern varied depending on the animal and matrix, but not on the maternal relationship. Despite compositional differences, the shared taxa reinforce the idea of interaction among the studied microbial communities.

## Graphical abstract

- Milk microbiomes showed similar composition in young and adult buffaloes.
- Despite distinct microbial profiles among matrices, shared bacterial families indicate microbial interaction between milk, faeces, calf spittle and soil.

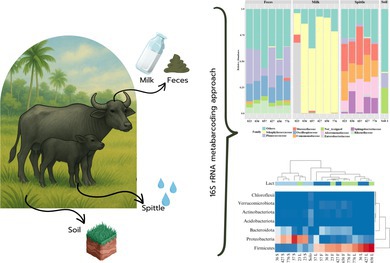

## 1 Introduction

The buffalo ( *Bubalus bubalis* ) is a livestock resource of great global importance, accounting for approximately 15% of the world's total milk production. Moreover, buffalo milk has attracted the attention of consumers and the dairy industry due to its superior nutritional characteristics, making it an ideal raw material for the production of cheese (such as Mozzarella) and yogurt (Guzman et al. 2020).

Compared to cow milk, buffalo milk has higher levels of total solids, fat and protein (the latter reaching up to 5.20 g/100 mL). In addition, it stands out for its micronutrient profile, being a matrix rich in bioactive components, with higher levels of Vitamin A (69 mg/100 g compared to 46 mg/100 g in cow milk) and biotin (13 mg/100 g compared to 2.0 mg/100 g in cow milk) (İpek 2024). Its calcium content is comparable, but with a notably lower sodium concentration (37 mg/100 g vs. 58 mg/100 g in cow's milk), a feature that may positively influence calcium retention in the human body (İpek 2024).

Milk, when synthesised and secreted by the mammary alveoli of a healthy animal, is considered sterile and the mammary gland should not contain bacteria. However, as it is excreted through the teat canal, it becomes colonised by a variety of microorganisms naturally present on the epithelial lining of the teat canal and in the surrounding environment (Melini et al. 2017; Ganda et al. 2016; Ding et al. 2025). The raw milk microbiome is highly diverse and dynamic, composed mainly of bacteria, followed by fungi and yeasts. In this context, raw buffalo milk presents a complex microbiota, including beneficial bacteria (such as lactic acid bacteria—LAB) as well as pathogenic microorganisms (such as *Pseudomonas*, which produces enzymes that degrade fat and protein) and foodborne pathogens such as *Listeria*, *Salmonella* and *E. coli* . In studies conducted by Ferrocino et al. (2021) using next‐generation sequencing (NGS) on raw buffalo milk, the genera *Lactococcus*, *Acinetobacter*, *Moraxella*, *Streptococcus*, *Anoxybacillus* and *Aeromonas* were consistently identified as dominant bacterial groups. According to Habiba et al. (2025), this diversity represents a promising resource for innovations in probiotic dairy products derived from buffalo milk.

The composition of milk and its microbiome can be influenced not only by using antimicrobials (Ganda et al. 2016), as commonly expected, but also by management systems, climatic conditions, diet and hygiene. The dairy farm environment is a potential source of contamination, with microorganisms originating from faeces, water, feed, bedding and air. Microbial source‐tracking studies (using the FEAST algorithm) in raw buffalo milk have confirmed that the teat surface and the teat cup liner are the main sources of microbial contamination, emphasising the importance of hygiene during the milking process (Miao et al. 2024).

Lactation stages and physiological status are also critical factors that influence the microbial composition of milk in species such as cows (Lima et al. 2018; Metzger et al. 2018), goats (McInnis et al. 2015) and buffaloes (Catozzi et al. 2020), suggesting that various external and physiological aspects may affect these communities. For example, the physiological status of the buffalo, such as the transition between lactation and the dry period, triggers microbial remodelling in the rumen to adapt energy and lipid metabolism to the demands of milk production, affecting the fatty acid composition of milk (Ding et al. 2025). Differences in the microbial composition of buffalo milk vary significantly among countries and regions due to environmental, climatic and genetic factors (Kable et al. 2016; Chaves Lopez et al. 2016), highlighting the importance of regional studies such as the one proposed here in the Marajó Archipelago.

In the context of the cow‐calf relationship, maternal contact in dairy production systems is a key factor for animal health and welfare, allowing the expression of natural behaviours, stimulating calf development and improving growth and weight gain rates (Johnsen et al. 2016). Moreover, milk as the sole postnatal food source is essential for the calf, serving as a matrix that actively modulates the intestinal microbiome and offers therapeutic and preventive potential against various health conditions (Ertürkmen 2025). Additionally, this contact positively affects udder health, mainly due to suckling, indicating a beneficial relationship between the milk microbiome, nursing and calf health (Johnsen et al. 2016).

Furthermore, calf behaviours such as grazing can lead to geophagy. Soil is a source of microorganisms, and previous studies have demonstrated a relationship between diet and rumen microbiome composition in cows and buffaloes. Therefore, it is suspected that microbial exchange may also occur through accidental soil ingestion (Iqbal et al. 2018; Noronha et al. 2023).

In the Marajó Archipelago, buffalo farming plays a crucial role in consolidating the local economy, especially considering the global trend of valuing regional products. Consumers are increasingly seeking unique flavours and more nutritious foods. In this context, despite the growing visibility of dairy buffalo farming and its derived products, there is still a lack of studies on the buffalo milk microbiome and its interactions with the calf and the environment.

Thus, we employed 16S rRNA metabarcoding to study the bacterial diversity of buffalo milk (from heifers and cows) and compare its composition to that of their faeces, their respective calves (spittle), and the environment (pen soil). In research settings, sequencing and analysis of hypervariable regions of the 16S rRNA gene are a widely trusted method for characterising the bacterial diversity of a given sample (Catozzi et al. 2020; Oikonomou et al. 2014). Furthermore, the hypothesis that there is microbial sharing among the matrices studied (milk, spittle, faeces and soil) was tested in the present study.

## 2 Materials and Methods

### 2.1 Animal Ethics and Genetic Heritage Management

The Animal Ethics Committee of the Federal Rural University of the Amazon approved this experiment under Protocol No. 6612040821 (CEUA) and 636‐202 (UFRA). The project was also registered with the Genetic Heritage Management Council of the Federal Government in the National System for the Management of Genetic Heritage and Associated Traditional Knowledge under registration number A144BC3. The data are available in the Sequence Read Archive (SRA) database under accession number PRJNA1111038.

### 2.2 Sampling Site

**FIGURE 1:**
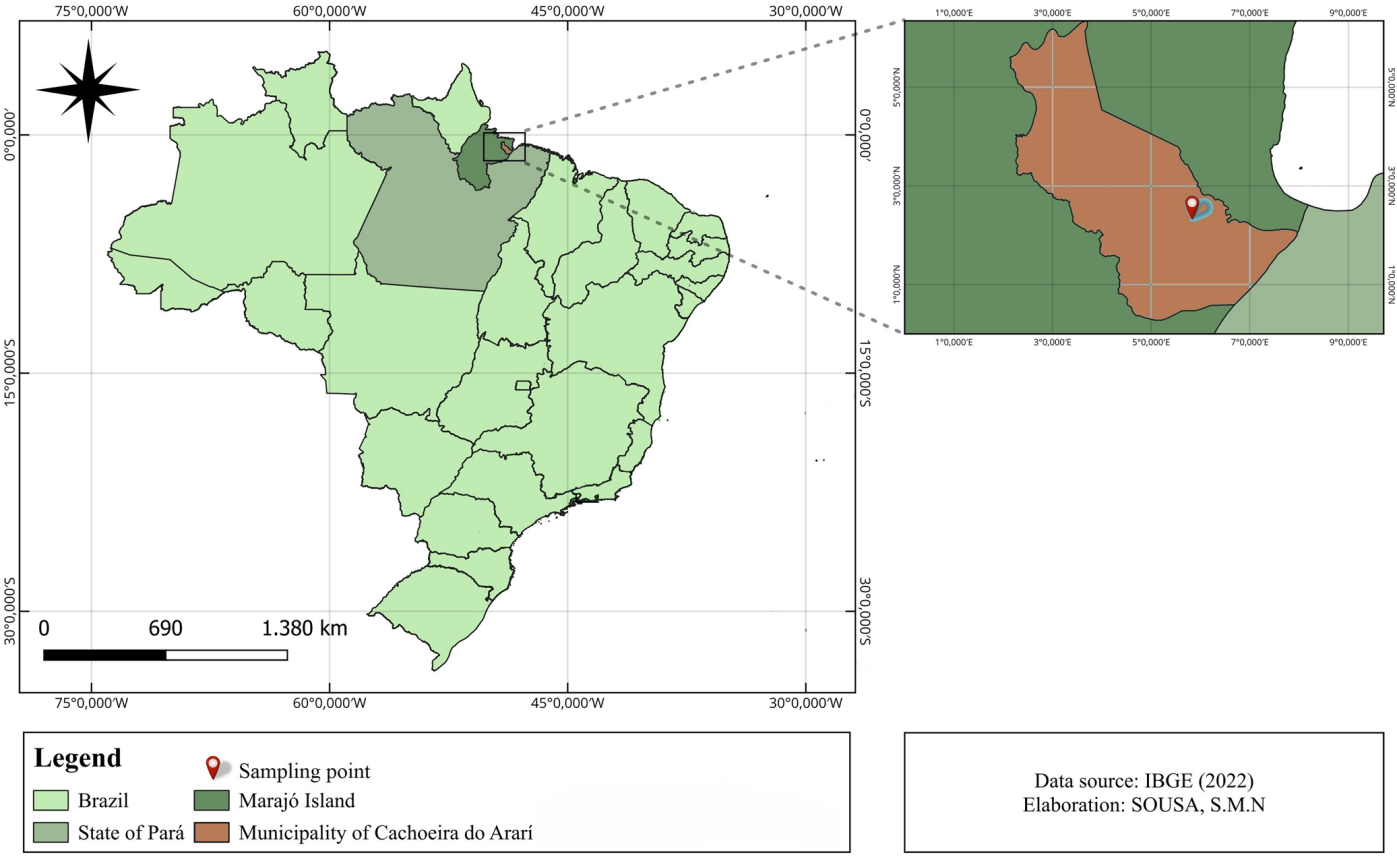
Location of the farm where the samples were collected, in the municipality of Cachoeira do Arari—Marajó Archipelago, Pará, Brazil.

Samples were collected in May 2023, during the rainy season, on a property located in the Marajó Archipelago, in the municipality of Cachoeira do Arari (Figure 1), in the state of Pará, Brazil.

### 2.3 Sample Collection

The herd on the property consisted of 120 crossbred buffaloes (Murrah and Mediterranean breeds). These animals were kept in the same paddock regardless of age and were milked twice a day, with an average daily milk yield per animal ranging from 6 to 10 L. Calves remained with their mothers for 2 h after milking and suckled residual milk.

A completely randomised experimental design was adopted. A total of 18 samples were collected: six milk samples and six faecal samples from healthy buffaloes, and six spittle samples from their respective calves. The animals were selected according to the following criteria: (i) Buffaloes during 1st lactation (*n* = 3) or 3rd lactation (*n* = 3); (ii) Animals with similar genetic backgrounds; (iii) Animals at the same stage of lactation; and iv. Animals under the same general, nutritional and milking management conditions.

Clinical examination of the mammary gland was performed (Grunert 1993; Radostits et al. 2009), and the presence of mastitis was evaluated using the strip cup test (Grunert 1993) and the California Mastitis Test (Schalm and Noorlander 1957). Antisepsis of the teats was then carried out with cotton soaked in 70% alcohol, the first three milk jets were discarded and milk was subsequently collected. For somatic cell count (SCC), milk was collected from each quarter and placed into tubes containing bronopol preservative, then sent to the Clínica do Leite Institute, where SCC was determined using flow cytometry (×1000/mL)—PO ANA 001:05, and results were analysed statistically using Tukey's test (*p* < 0.05).

Bacterial DNA was extracted from pooled milk samples from all four quarters of each animal, resulting in one composite sample per animal. All milk samples were collected in the morning, before regular milking, under standardised conditions for all animals, in order to minimise potential diurnal variation effects on the microbiome composition.

Faecal samples were collected by spontaneous defecation, calf spittle was collected using a syringe to stimulate spittle production, and soil samples were obtained as a composite from various points in the paddock where the animals were kept. All samples were placed in sterile containers, refrigerated in an insulated box with reusable ice packs, and transported to the Center for the Valorization of Bioactive Compounds of the Amazon (CVACBA) for bacterial DNA extraction.

### 2.4 DNA Extraction and Sequencing of the 16S rRNA V4 Region

Total DNA was extracted from milk after fat removal was performed following Oikonomou et al. (2014). All extractions were carried out using the PureLink Genomic DNA Purification Kit (Invitrogen, Carlsbad, CA, USA), according to the manufacturer's instructions. DNA samples were quantified using a Qubit 4.0 fluorometer (Thermo Scientific, Waltham, MA, USA) to assess purity and concentration, and then stored at −20°C.

Sequencing of the 16S rRNA genes began with amplification of the hypervariable V4 region using Illumina‐tailed primers: V4 515F (5′ TCGTCGGCAGCGTCAGATTGTATAAGAGACAGGTCCAGCMGCCGGGTAA 3′) and V4 806R (5′ GTCTCGTGGGCTCGGAGATGTGTATAAGAGACAGGGACTACHVGGGTWTCTAAT 3′). PCR reactions were performed in a final volume of 20 μL, containing 6 μL of DNA (12 ng/μL), 2 μL of each primer and 10 μL of Phusion Master Mix.

Two PCRs were carried out: the first to amplify the V4 region of the 16S gene, and the second to index the generated fragments. The first PCR consisted of 25 cycles, while the indexing PCR had 8 cycles. Amplification conditions for both reactions were: initial denaturation at 98°C for 30 s, followed by cycles of 98°C for 30 s, 55°C for 60 s and 72°C for 30 s, with a final extension at 72°C for 5 min. Resulting amplicons were purified, pooled, quantified and sequenced using the Illumina MiSeq platform with the v2 nano kit (Illumina, San Diego, CA, USA; 300 cycles), paired‐end reads (2 × 250 bp), following the manufacturer's instructions.

### 2.5 Data Analysis

The 16S rRNA V4 region data analysis followed several steps to ensure accuracy. Initially, Forward (R1) and Reverse (R2) read files were renamed according to the final naming standard. Quality graphs were generated using the R software (available at https://www.r‐project.org) to assess sequencing quality before any filtering or trimming.

Raw microbiota data were processed using the Divisive Amplicon Denoising Algorithm (DADA2) version 316, as described by Callahan et al. (2016). The resulting reads were merged, and chimeric sequences were removed to ensure data integrity. Taxonomic assignment was carried out using the Amplicon Sequence Variant (ASV) method, based on the SILVA database (Silva_nr_v128) (Mcmurdie et al. 2013).

Alpha and beta diversity analyses were conducted in R v.4.3.1 using the phyloseq v.1.22.3 (Oksanen et al. 2018) and vegan v.2.6‐4 (“CRAN ‐ Package vegan,” 2022; 2018, p. phyloseq) packages (Chi et al. 2019). Alpha diversity indices such as Chao, Shannon and Simpson were calculated and compared using PERMANOVA and Kruskal‐Wallis tests. Bray‐Curtis, Unifrac and Weighted Unifrac distances were used for beta diversity analysis after removing rare taxa (< 1%). Other plots, such as rarefaction curves, relative abundance and Venn diagrams, were generated using default settings from the respective R packages.

To analyse shared microbial families, the packages phyloseq, VennDiagram and RColorBrewer were used within the RStudio environment. Sequencing data were processed and grouped using the Phyloseq package (Mcmurdie et al. 2013) at the Phylum, Order and Family taxonomic levels. Then, the get_vennlist function from the MicrobiotaProcess package was used to create lists of families present in each sample group (Faeces, Milk and Spittle). To visualise intersections among the different sample groups, the VennDiagram package (Chen and Boutros 2011) was used.

## 3 Results

### 3.1 Somatic Cell Count

**FIGURE 2:**
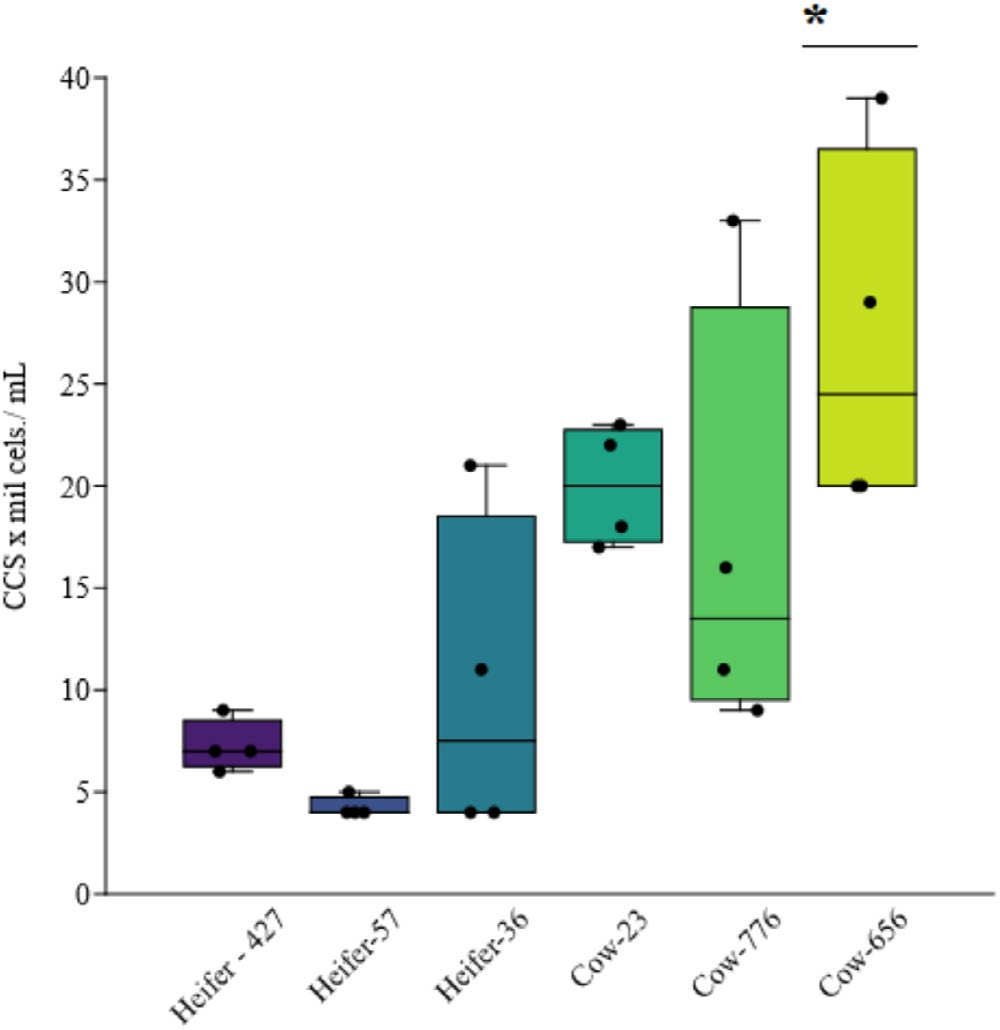
Somatic cell count (SCC) in milk from cows and heifers. (•) indicates the distribution of SCC values per mammary quarter for each animal evaluated. (—) represents the average SCC value per animal. Represented by: Heifers (036, 057 and 427) and cows (023, 776 and 656). *p < 0.05.

All milk samples investigated were free of mastitis as shown by macroscopic examination of the secretion and the California Mastitis Test. However, significant differences in somatic cell count were observed among the different groups analysed (ANOVA, F = 5.461, *p* = 0.001). Notably, significant differences were found between Cow‐656 and Heifer‐427 (Tukey; *p* = 0.018), Cow‐656 and Heifer‐57 (Tukey; *p* = 0.006) and Cow‐656 and Heifer‐36 (Tukey; *p* = 0.049), as shown in Figure 2.

### 3.2 Alpha and Beta Diversities

A total of 2,743,330 raw 16S rRNA gene sequences were analysed. After quality assessment and trimming, the average number of sequences per sample was 20,003. The highest number of sequences per sample was observed in samples 427‐L and 036‐L, with over 30,000 reads, while the remaining samples ranged from 16,000 to 27,000 reads. A total of 3882 ASVs were obtained and compared using the SILVA database for taxonomic assignment (Table S1).

Alpha diversity results for species richness (Observed), diversity (Shannon) and Simpson indices are presented in Table S1. The observed metric showed that spittle samples exhibited greater species richness compared to faeces and milk samples. Shannon indices for the spittle matrix ranged from 4.59 to 4.97, whereas faecal samples ranged from 4.18 to 4.50, and milk samples from 3.43 to 3.88, indicating a diversity of species in the different sample types. Simpson index values were close to 1, suggesting no dominant bacterial communities within the samples.

The rarefaction curve showed a different trend depending on the sample matrix. In this study, spittle samples demonstrated the highest richness, followed by faeces and milk samples, respectively, which behaved similarly. All rarefaction curves reached a plateau regardless of the number of reads per sample (Figure S1).

**FIGURE 3:**
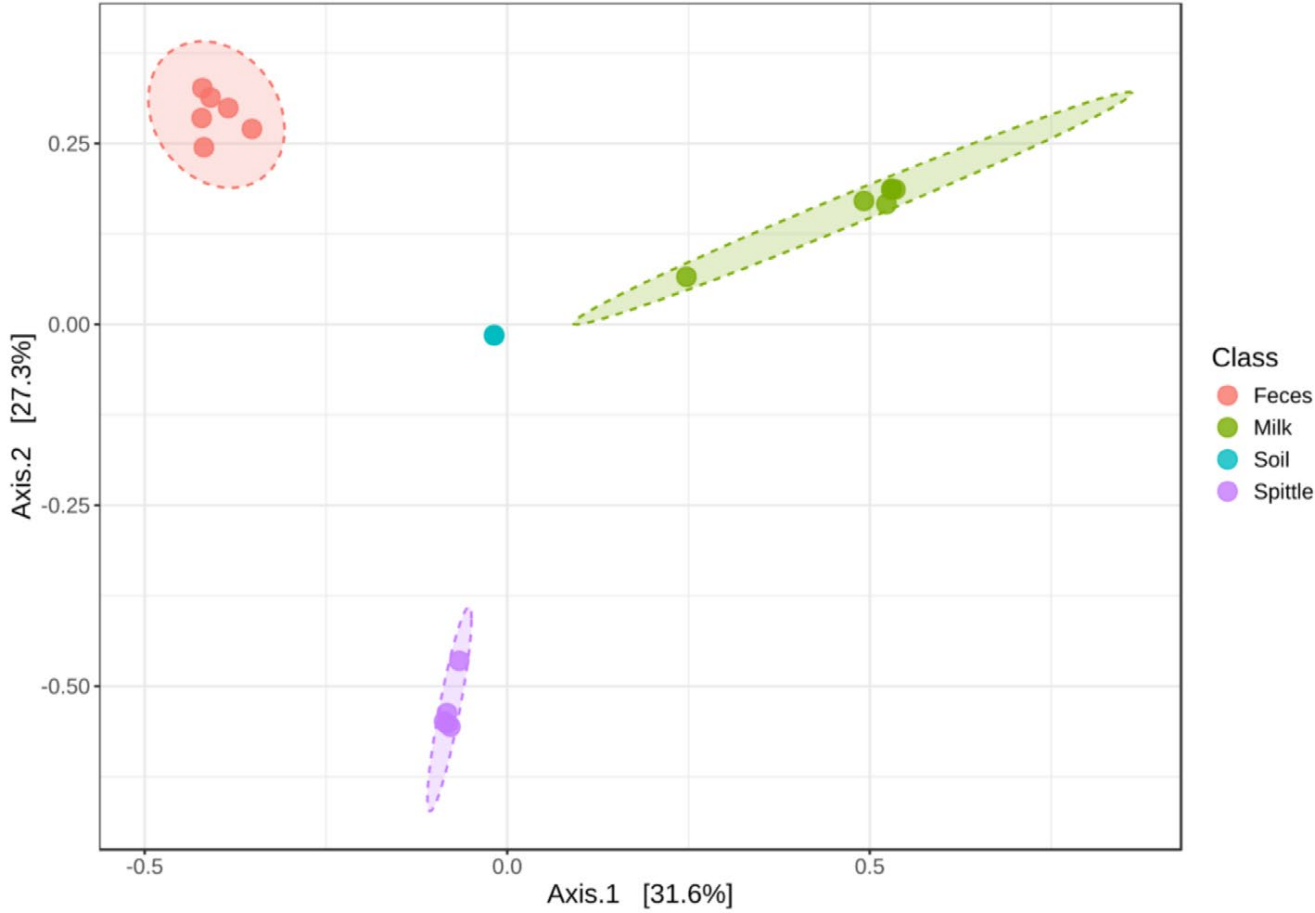
Graphical representation of beta diversity through principal coordinates analysis (PCoA), calculated using the taxon abundance matrix from faecal and milk samples of first‐ and third‐lactation buffaloes, spittle from their calves, and soil. The plot model explains 67.6% of the total data variance and R‐squared: 0.78553 and *p* = 0.001.

Similarity analysis between the studied matrices and the principal component analysis using the Unifrac metric—which considers both taxa and their respective abundance—revealed differences between matrices and identified distinct clusters based on microbiome profiles. All milk samples clustered in the first quadrant, with one of them closely positioned near the soil sample, which was located in the second quadrant along with all faecal samples. No sample from any matrix appeared in the third quadrant. In contrast, all spittle samples are clustered together in the fourth quadrant, separately from the others (Figure 3).

These results indicate that the community structures—both phylogenetic and ecological—of the faecal samples and one milk sample were similar to that of the soil sample. Meanwhile, spittle samples derived from calves were distinct from milk and faeces samples from buffaloes and from soil samples. This separation is possibly related to the greater contact calves have with the soil, either by ingesting it or lying on it.

### 3.3 Taxonomic Composition of Microbial Communities

**FIGURE 4:**
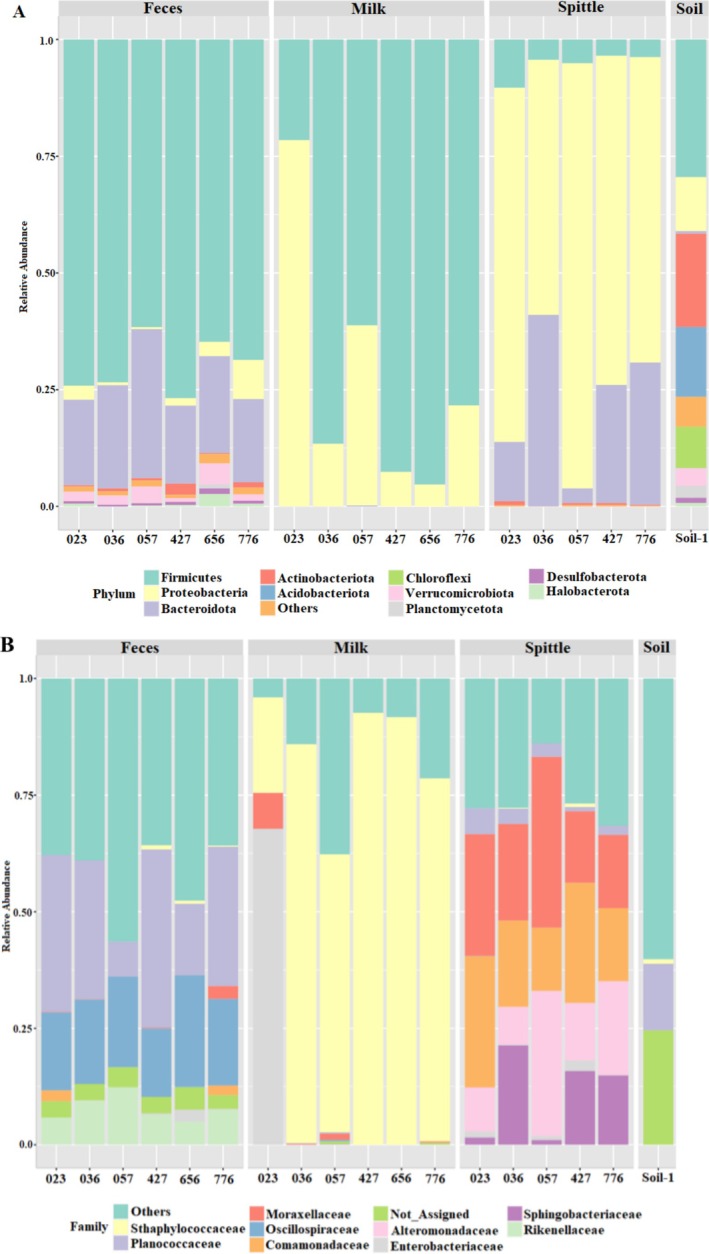
Bar plots showing the relative abundance of 16S rRNA gene amplicon reads assigned at the phylum (A) and family (B) levels, for faecal and milk samples from first‐ and third‐lactation animals, spittle from their respective calves, and soil where they were kept. Represented by: Heifers (036, 057 and 427) and cows (023, 776 and 656). Unclassified ASVs are shown as “Not_Assigned.” The 11 most representative taxa are presented in the legends. Each sample ID represents an individual animal. No technical replicates were used, and statistical analyses were conducted considering only biological replicates.

A total of 35 phyla, 79 classes, 148 orders and 208 families were identified from the analysed dataset. The most abundant phyla observed were *Firmicutes*, *Proteobacteria*, *Bacteroidota* and *Actinobacteriota* (Figure 4A). *Firmicutes* and *Bacteroidota* were predominant in faecal samples; *Firmicutes* and *Proteobacteria* in milk samples; and *Bacteroidota* and *Proteobacteria* in spittle samples. The soil sample was dominated by *Firmicutes* and *Actinobacteriota*. These findings highlight shifts in bacterial composition across all matrices studied.

Regarding bacterial classes, *Bacilli*, *Gammaproteobacteria*, *Clostridia* and *Bacteroidia* were the most abundant. *Clostridia* predominated in faeces, while *Bacilli* were more prevalent in milk and soil, and *Gammaproteobacteria* stood out in spittle. The most abundant bacterial taxa were affiliated with the orders *Staphylococcales*, *Bacillales*, *Oscillospirales*, *Enterobacterales*, *Burkholderiales* and *Pseudomonadales*, with *Oscillospirales* and *Bacillales* more prevalent in faeces; *Staphylococcales* and *Burkholderiales* in milk; and *Pseudomonadales* and *Burkholderiales* in spittle, respectively.

Figure 4B shows that the family *Planococcaceae* was predominant in faecal samples; *Staphylococcaceae* was more abundant in most milk samples, with *Enterobacteriaceae* prevailing in only one milk sample; and *Moraxellaceae* was the most abundant in spittle samples. At the genus level, *Ruminococcaceae*, *Rheinheimera* and *Kurthia* were predominantly found in faecal samples; *Staphylococcus*, *Burkholderia* and *Pseudomonas* were most frequently observed in milk samples, along with a slight presence of *Ruminococcaceae*; while *Acinetobacter*, *Chishuiella* and *Rheinheimera* were the most prominent genera in spittle samples.

**FIGURE 5:**
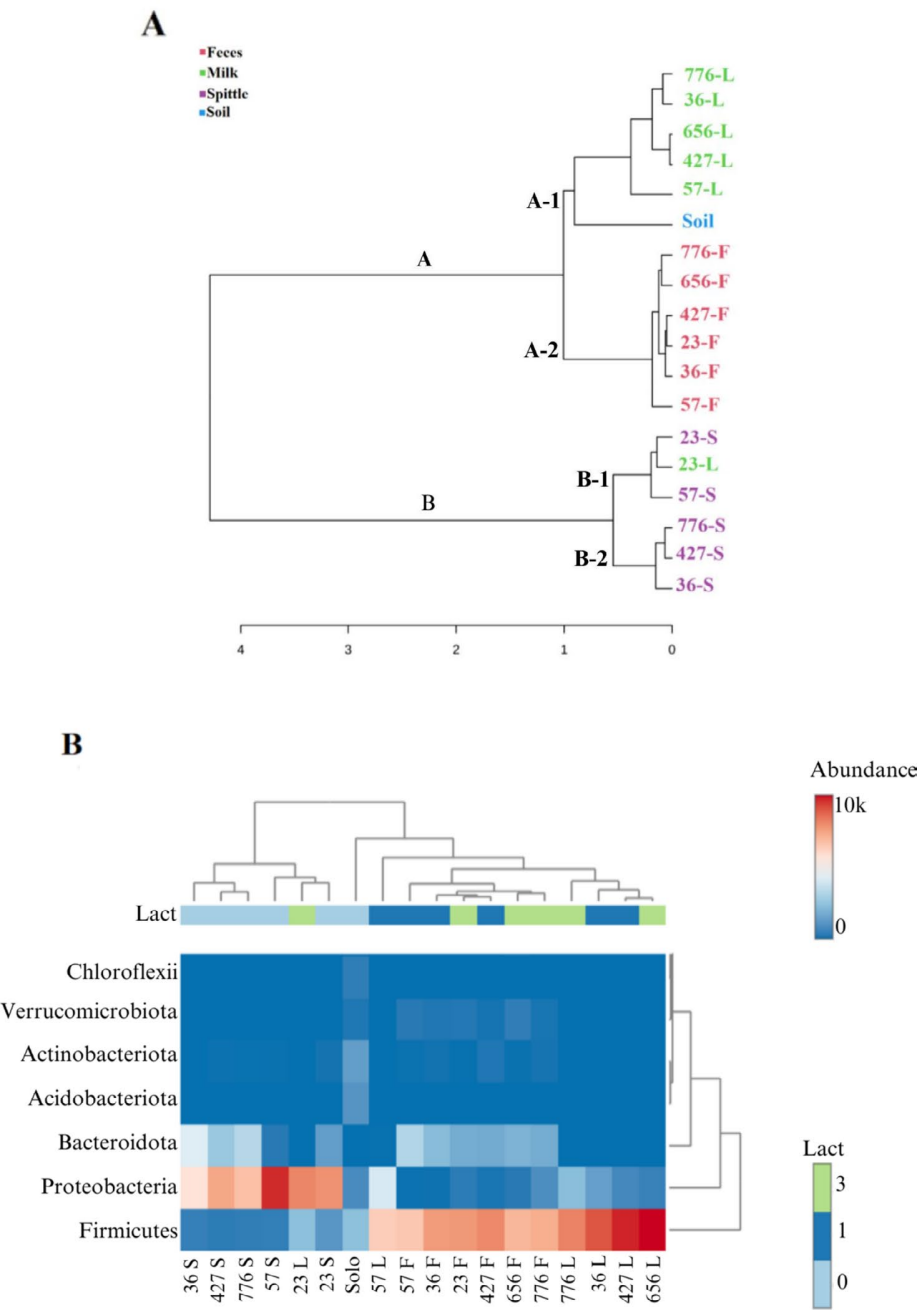
(A) Dendrogram based on hierarchical clustering of the relative abundance of ASVs for each sample type (faeces, milk, spittle and soil). (B) Heatmap demonstrating the relative abundance of phyla according to the sample matrix and classifications: Lact. *Lact‐ number of lactations*. Represented by: Heifers (036, 057 and 427) and cows (023, 776 and 656).

Cluster analysis by composition similarity revealed the formation of two distinct clusters: A and B. Cluster A was subdivided into A‐1 and A‐2. A‐1 contained all milk samples, with the soil sample positioned nearby, and A‐2 contained all faecal samples. Cluster B was subdivided into B‐1 and B‐2, where B‐1 contained two spittle samples and one milk sample, while B‐2 consisted of the remaining spittle samples (Figure 5A).

The heatmap, represented by colour intensity, shows differences in phylum abundance across the studied matrices. The phylum *Firmicutes* was the most abundant in all but one milk sample and all faecal samples, while *Proteobacteria* was the most abundant phylum in all spittle samples and one milk sample (from cow 023). *Bacteroidota* was also present in both spittle and faeces. Phyla such as *Acidobacteriotia*, *Actinobacteriota*, *Verrucomicrobiota* and *Chloroflexi* were less abundant but more prominent in the soil sample and the faecal samples from buffaloes (Figure 5B).

**FIGURE 6:**
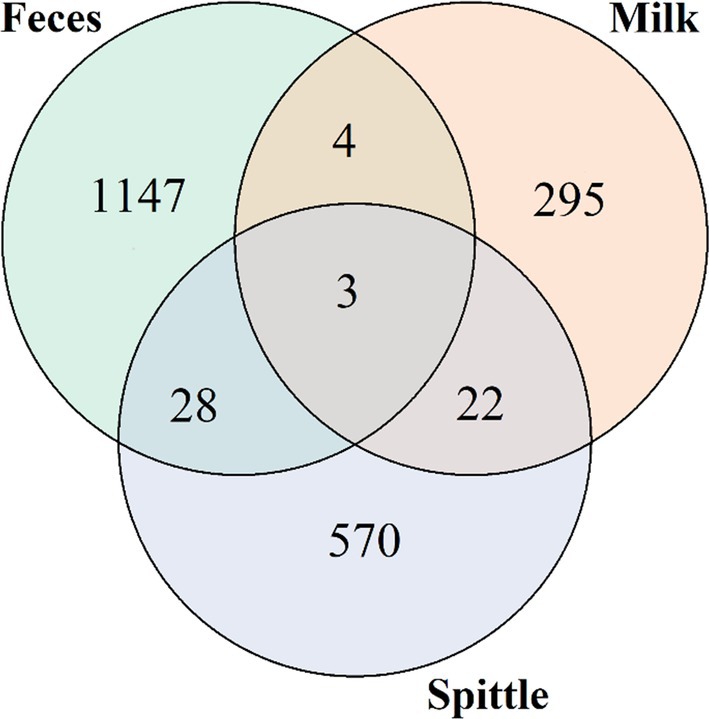
Venn diagram showing the number of individual and shared ASVs from the analysis of the microbiomes of faeces and milk from buffalo cows and spittle from their respective calves.

To describe the general composition of the microbiome in the matrices of buffalo milk, faeces and spittle, we used ASV taxonomy data to construct a Venn diagram (Figure 6). This analysis revealed the presence of 3511 ASVs obtained from the samples studied. Among the ASVs present in each matrix individually, 1147 were from faeces, predominantly from the phylum *Firmicutes* (54.4%), followed by *Bacteroidetes* (27.29%), *Verrucomicrobia* (3.75%), *Proteobacteria* (2.79%), *Actinobacteria* (2.01%) and others (9.76%); 270 ASVs were from milk samples, predominantly from *Proteobacteria* (60.68%), *Firmicutes* (34.92%), *Actinobacteria* (2.03%), *Bacteroidetes* (1.69%), *Chloroflexi* (0.34%) and others (0.34%); and 2094 ASVs from spittle samples containing predominantly *Proteobacteria* (56.49%), followed by *Bacteroidetes* (27.89%), *Firmicutes* (12.46%), *Actinobacteria* (2.11%), others (0.7%), as well as the phylum *Deinococcus‐Thermus* (0.35%).

When ASVs shared between matrices were evaluated, we found three ASVs present in all matrices; four were shared between faeces and milk, belonging to the phyla *Firmicutes* and *Proteobacteria*; 22 were shared between milk and spittle, belonging only to the phylum *Proteobacteria*; and 28 were shared between faeces and spittle, belonging to the phyla *Proteobacteria* and *Firmicutes*.

**FIGURE 7:**
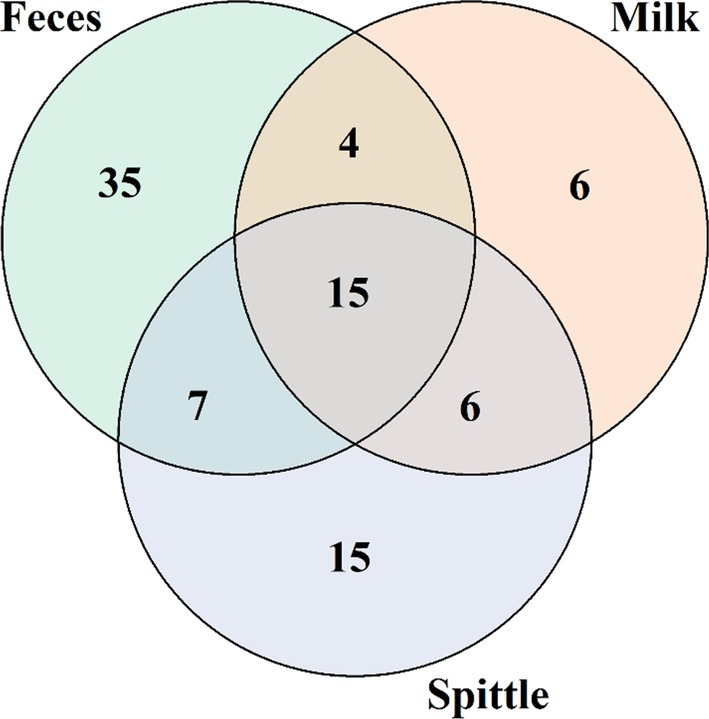
Venn diagrams demonstrating the sharing at the family level between matrices through alpha diversity.

Based on the relative abundances found in the alpha diversity analysis, a Venn diagram was created to demonstrate the results of taxon sharing at the family level in greater depth. Figure 7 shows that 15 families were shared among the three matrices, namely: *Staphylococcaceae*, *Planococcaceae*, *Enterobacteriaceae*, *Moraxellaceae*, *Ruminococcaceae*, *Pseudomonadaceae*, *Flavobacteriaceae*, *Lachnospiraceae*, *Xanthomonadaceae*, *Porphyromonadaceae*, *Peptostreptococcaceae*, *Prevotellaceae*, *Caulobacteraceae*, *Micrococcaceae* and *Rhodobacteraceae*. Six families, namely *Burkholderiaceae*, *Aeromonadaceae*, *Sphingobacteriaceae*, *Pasteurellaceae*, *Oxalobacteraceae* and *Propionibacteriaceae*, were shared only between milk and spittle. Milk and faeces shared *Christensenellaceae*, *Alcaligenaceae*, *Methylobacteriaceae* and *Paenibacillaceae*. And faeces and spittle shared *Comamonadaceae*, *Bacteroidaceae*, *Clostridiaceae*, *Microbacteriaceae*, *Campylobacteraceae*, *Rhizobiaceae* and *Family_XI*.

**FIGURE 8:**
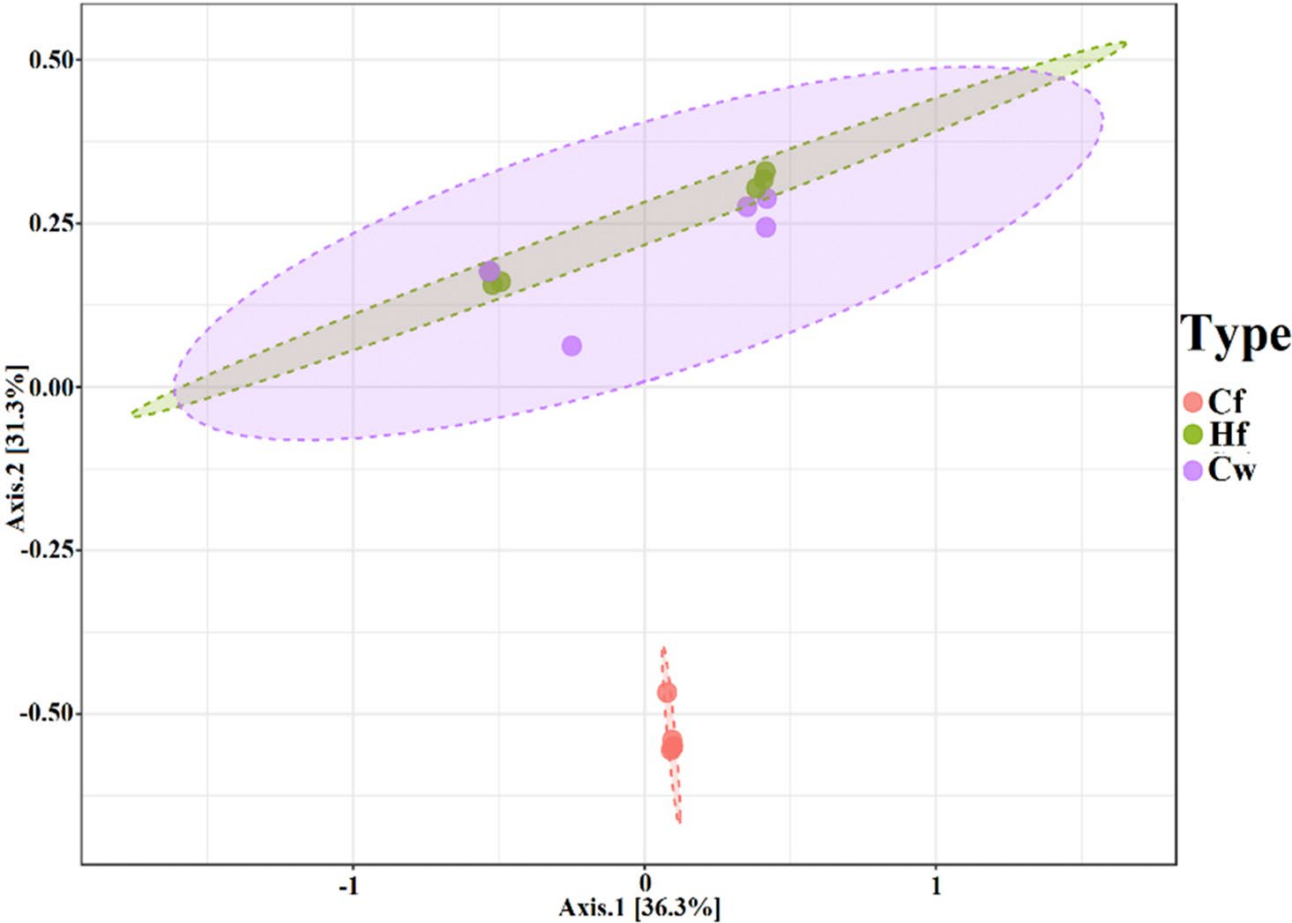
Graphical demonstration of beta diversity analyses—weighted Unifrac of milk and faecal samples from first‐lactation (Hf) and third‐lactation (Cw) buffaloes, and spittle from their calves (Cf). The plot model explains 67.4% of the total data variance and R‐squared: 0.8153 and *p* = 0.002.

The analysis of the biological samples from the buffaloes resulted in the grouping of all milk samples, both from first‐lactation heifers (Hf) and third‐lactation cows (Cw). This result demonstrates that, despite the significant difference in somatic cell counts between young and adult animals, when similarity and principal component analysis are observed, they appear to be grouped. The same was observed among the faecal samples from these animals and the spittle from their calves (Figure 8).

Thus, the somatic cell count associated with alpha diversity indices identified Pearson correlation values of up to −0.56, suggesting an inversely proportional correlation (Figure S2).

Microbial sharing among the studied matrices was statistically investigated by PERMANOVA analysis and revealed significant differences in microbial composition between milk, faeces, spittle and soil samples (*F* = 8.42, *R* ^2^ = 0.65, *p* = 0.001). Similarity analysis (ANOSIM) confirmed these differences (*R* = 0.78, *p* = 0.001), indicating dissimilarity between groups. Taxonomic sharing was quantified by applying a chi‐squared test to the 15 families shared among all matrices and revealed a significant association (*χ* ^2^ = 142.3, df = 42, *p* < 0.001). Indicator species analysis (IndVal) identified *Staphylococcaceae* as a significant indicator of milk (IndVal = 0.85, *p* = 0.002), *Planococcaceae* of faeces (IndVal = 0.72, *p* = 0.008) and *Moraxellaceae* of spittle (IndVal = 0.68, *p* = 0.012).

Regarding the effect of age on the microbial composition of milk, contrary to the differences observed in somatic cell counts, PERMANOVA detected no significant differences between heifers and cows (*F* = 1.23, *R* ^2^ = 0.14, *p* = 0.312). This lack of age‐related differentiation was corroborated by alpha diversity indices, which also did not differ significantly between age groups (Shannon: *t* = −0.85, *p* = 0.435; Simpson: *t* = −0.42, *p* = 0.689; Chao1: *t* = −1.12, *p* = 0.308), demonstrating that despite physiological differences related to age, the structure of milk microbial communities remains similar between young and adult animals.

## 4 Discussion

SCC is an important indicator of milk quality as well as udder health. SSC by flow cytometry as used in this analysis stands out for being precise and highly reproducible and, therefore, has been standardised by international dairy product organisations (Catozzi et al. 2017). Previous studies conducted on buffaloes showed that a healthy mammary gland can release up to 200,000 cells/mL in milk (Mesquita et al. 2017) In the present study heifers presented between 7000 and 21,000 cells/mL and cows between 9000 and 39,000 cells/mL, thus reflecting the good health of the mammary quarters of these animals. In our study, we observed an increase in somatic cell counts in older cows. This increase may be partly attributed to exposure to microorganisms throughout their life, stimulating their immune system and modulating the local microbiome, highlighting the importance of considering age and clinical history when evaluating milk quality and udder health in buffaloes (Catozzi et al. 2020).

In the present study, the microbiomes of buffalo milk and faeces, calf spittle and the environment were investigated using 16S rRNA gene sequencing. Due to its high throughput, 16S rRNA sequencing has become a widely used tool, replacing approaches such as microorganism culturing, and has become the method of choice for research on bacterial community structure and diversity (Yang et al. 2024). Despite the growing visibility of buffalo production, previous studies have analysed the metabolome of buffalo milk (Salzano et al. 2020) and the behaviour of the microbiome during mastitis (Catozzi et al. 2020), but only one study has been conducted to thoroughly investigate the buffalo milk microbiome (Luziatelli et al. 2023). To the best of our knowledge, no microbial ecology study involving milk, faeces and calf spittle has been carried out in buffaloes from the Marajó Archipelago region or in other regions of Brazil.

In this work, a plateau was observed in the rarefaction curve, indicating that a high coverage of the species diversity present in the samples was captured by sequencing. This was also observed in previously published studies, where increasing sequencing depth also increased the number of ASVs or species detected, leading to this plateau (Chi et al. 2019).

Soil exhibited higher microbial taxonomic diversity than the other matrices analysed, which is justified by being a matrix that harbours a highly heterogeneous microbiome, with more than 50,000 species per gram (Fierer 2017; Banerjee and Van Der Heijden 2023). The interaction between soil microbiome and animal intestinal microbiome is well‐documented, and estimates suggest that up to 3% of the ruminal microbiome in sheep and cattle may be contributed by ingested soil (Liddicoat et al. 2019). Buffaloes, like cattle, are also animals with low epicritic sense, which causes accidental ingestion of soil in their diet, and this could explain the observed sharing of ASVs between soil and the other matrices.

The results show that *Staphylococcaceae* is the main family of the Firmicutes phylum present in milk samples, which includes common components of the milk and skin microbiome of mammals. Following that, the families *Enterobacteriaceae* and *Moraxellaceae*, which belong to the *Proteobacteria* phylum, were found. This study aligns with previous studies (Catozzi et al. 2017; Salman et al. 2023) that observed similar results at phylum and family levels (Catozzi et al. 2017), where the highest frequency of *Staphylococcaceae* and *Moraxellaceae* families was identified in milk samples from healthy animals. The abundance of these specific phyla in cows varied with seasonality and the positive effect of higher humidity and temperature (Celano et al. 2022). This may be directly related to their abundance in the sampling used in the present study, considering the hot and humid climate in the Amazonian biome. Despite being described in milk from healthy buffaloes, the *Moraxellaceae* family was mentioned in another study for including psychrotrophic bacteria, which can appear in the environment, with some known for their ability to form biofilms on various surfaces (Maes et al. 2019), consequently causing changes in milk properties. However, discrepancies were found with other previously published studies at the family level, where the authors identified the main families as *Corynebacteriaceae* and *Lactobacillaceae* (Luziatelli et al. 2023) and *Aerococcaceae* and *Corynebacteriaceae* (Catozzi et al. 2019), respectively. These variations may therefore be related to different farm management conditions, particularly in dairy production practices and their impact on milk composition, with the farm studied in this work adopting more rustic practices compared to those assessed in other studies.

The taxonomic composition analysis identified an unexpected abundance of *Enterobacteriaceae* in a milk sample from an apparently healthy animal. Previous studies have revealed that genera such as *Enterococcus*, *Klebsiella* and the species *Escherichia coli* , which belong to this family, are associated with subclinical mastitis (Catozzi et al. 2017), showing that sequencing analysis provides insights into the microbiological quality of milk and the health status of animals. In our study, the analysis of taxonomic composition revealed that the milk sample from cow 023 exhibited a distinct microbial profile compared to the other samples in the group, characterised by a disproportionately high abundance of the family *Enterobacteriaceae* (> 60% of relative abundance) compared to the other milk samples, which showed predominance of *Staphylococcaceae*. This sample was considered an outlier in subsequent analyses but was retained in the dataset due to the limited sample size and the importance of documenting natural microbial variability in Amazonian buffaloes. The elevated presence of *Enterobacteriaceae* may suggest environmental contamination or the onset of a subclinical infectious process, highlighting the importance of continuous monitoring of milk microbiological quality even in clinically healthy animals.

In the faecal microbiome of buffaloes, the family Planococcaceae is the most abundant, followed by Oscillospiraceae. Both families belong to the Firmicutes phylum. However, previous studies have shown that, although Firmicutes is the most abundant phylum, at the family level, the highest abundance was observed in Ruminococcaceae and Bacteroidaceae (Nguyen et al. 2020). In a study conducted on buffalo faeces (Sharma et al. 2021), the most abundant families found were Muribaculaceae and Christensenellaceae when the animals were evaluated during estrus, and Bacillaceae and Peptomicrobiaceae were found in higher abundance during diestrus and proestrus, respectively. Studies demonstrate that the intestinal microbiota is an important factor in host physiology, as numerous physiological and pathological processes are linked to it (Pluznick 2014).

In calves, the spittle microbiome plays a key role in the development of the gastrointestinal tract, food digestion, protection against pathogens and strengthening the immune system. The composition of the microbiome varies according to diet, age, breeding conditions and animal health (Mojahedi et al. 2018). When analysing the most prevalent families in calf spittle, we observed Moraxellaceae and Comamonadaceae, both belonging to the Proteobacteria phylum, which was the most abundantly observed. A study of the human spittle microbiome of healthy patients also found a predominance of the Proteobacteria phylum, supporting the findings of the present study (Soriano‐Lerma et al. 2020). Regarding the family, Moraxellaceae includes bacteria previously described in the milk of healthy animals and microorganisms present in soil and water (Luziatelli et al. 2023). The Comamonadaceae family has been recorded as part of the respiratory tract of primates (Facioli et al. 2020).

From the dendrogram constructed with the data studied, an initial animal‐dependent grouping was observed, where samples from buffaloes clustered together, regardless of the matrix and samples from calves formed a separate cluster. Additionally, no grouping between generations was observed, that is, between mothers and their first or third‐lactation offspring. Following that, a matrix‐dependent grouping was observed; however, despite an overall matrix‐dependent grouping, sharing of taxa between the matrices was noted.

Although only one soil sample was analysed in this study, upon observing the dendrogram and heatmap, this sample grouped more closely with the buffalo samples (milk and faeces) than with the calf samples (spittle). This may be related to the contact of adult animals with the soil through their diet and the contact of their mammary glands when lying on the ground. In the heatmap, the Firmicutes phylum was prominent in the buffalo samples (milk and faeces), which could be related to the higher abundance observed of the genera *Staphylococcus* (milk) and *Ruminococcaceae* (faeces), respectively. On the other hand, Proteobacteria were prominent in the calf samples (spittle), which could be associated with the higher abundance of the genera *Acinetobacter* and *Rheinheimera* in the spittle samples and *Burkholderia* in the milk sample.

When evaluating the sharing of the non‐cultivable microbiota from milk, spittle and faeces samples, a total of 15 families were identified at the family level, with two of them specifically showing high abundance in milk samples: Staphylococcaceae and Enterobacteriaceae, which, as mentioned earlier, include microorganisms commonly found in the milk microbiome of various species. Moraxellaceae, in turn, is a family with high abundance in both milk and spittle, supporting the idea of significant microorganism exchange between the buffalo and its calf through breastfeeding. Planococcaceae was shared among all three matrices, but its abundance was observed mainly in faeces. The increased abundance of this family has been positively correlated with dysbiosis caused by gastrointestinal inflammation due to *Mycobacterium avium* subsp. *paratuberculosis* in calves (Derakhshani et al. 2016) and in dry cows (Elmagzoub et al. 2024).

In our analysis, families shared only between milk and faeces of buffaloes were *Christensenellaceae* and *Paenibacillaceae* (both belonging to the Firmicutes phylum), and *Alcaligenaceae* and *Methylobacteriaceae* (belonging to the Proteobacteria phylum). Among these, *Christensenellaceae* stands out due to the presence of genera capable of producing a wide range of probiotic metabolites and secondary bile acids, benefiting the ruminal microbiota and improving food fermentation in these animals (Wei et al. 2022). This supports the findings of Young et al. (2015), who identified a route for microorganism exchange between the gastrointestinal system and the mammary gland of dairy cows via the bloodstream. In a study by Derakhshani et al. (2018), the increase of *Alcaligenaceae* in the colostrum of dairy cows was related to a decrease in richness and diversity indices, suggesting a possible dysbiosis in these animals.

In our study, six families were shared between milk samples from buffaloes and spittle from their calves. Preliminary studies suggest that the microbiota of calves is influenced by the type of contact, whether prolonged or not, between mother and offspring, resulting in the vertical sharing of microorganisms through milk ingestion (Wenker et al. 2022). Among the shared families, *Burkholderiaceae*, *Oxalobacteraceae* and *Propionibacteriaceae* have been described as components of the bovine milk microbiome (AoDaohu et al. 2024). However, Gathinji et al. (2023) described the relationship between the abundance of these families and higher levels of non‐esterified fatty acids, haptoglobin and aspartate transaminase in the plasma of dairy cows, suggesting that this abundance may be related to metabolic stress caused by the transition period from pregnancy to lactation in these animals. The family *Sphingobacteriaceae* was recently described to show a normal increase in the spittle of dairy calves aged 78–90 days (Amin et al. 2021), which is related to dietary changes during the early weaning phase. This may also occur with the buffalo calves in this study, as they are 60–75 days old and have started mixed feeding with free forage, in addition to milk intake.

Looking at families shared between spittle and faeces in this study, *Comamonadaceae* was found as the most abundant family present in the spittle. It is known that buffaloes, when feeding on forage, accidentally ingest soil and its contents, and as a result, microorganisms from faeces coexist in their spittle, including some representatives of the *Bacteroidaceae* family, which was found to have 6% abundance in the faecal microbiome of cows in a study by AoDaohu et al. (2024). *Rhizobiaceae*, on the other hand, is related to plants and is not a commensal of animals, but it is primarily associated with the ingested forage, and its presence in both faeces and spittle is concurrent. Amin et al. (2021) described an abundance of *Clostridiaceae* in the post‐weaning ruminal liquid microbiota of dairy calves, correlated with the ingestion of forage and mixed forages.

The statistical confirmation of microbial sharing among matrices, evidenced by the significance of the chi‐square test (*p* < 0.001), supports the existence of microbial transmission pathways, corroborating the hypothesis of interaction among microbial communities. This finding aligns with studies in cattle that demonstrated the transfer of intestinal bacterial components to mammary secretions via the bloodstream (Young et al. 2015). The identification of specific indicator families for each matrix (Staphylococcaceae in milk, Planococcaceae in faeces and Moraxellaceae in spittle) suggests that, although sharing occurs, each niche maintains distinct microbial characteristics which are determined by specific physiological and environmental conditions. The statistical significance of this sharing indicates that microbial interaction is not merely random but represents fundamental biological processes of microbial transfer and community establishment.

The absence of a significant correlation between maternal and offspring microbial profiles, as shown by the Mantel test (*r* = 0.12, *p* = 0.284), contrasts with studies reporting vertical transmission of microorganisms in other production systems (Wenker et al. 2022). This pattern may reflect the dietary transition of calves, which started consuming forage in addition to milk intake, thus substantially altering their oral microbial environment (Amin et al. 2021). These findings suggest that environmental and management factors exert a predominant influence over maternal microbial inheritance in the age range of the calves studied (60–75 days). The significant differentiation observed in the PERMANOVA between maternal and calf samples (*p* = 0.001) reinforces that, although some microbial families are shared, the communities are structured in an animal‐dependent manner.

It is important to highlight that the present study included a limited number of animals per group, which naturally constrains the statistical power of the comparisons. This choice was motivated by logistical aspects and the exploratory nature of the research. Nevertheless, high‐throughput sequencing generated a large amount of data per sample (Figure S3), enabling comprehensive coverage of microbial diversity. Still, we acknowledge that future studies should prioritise greater biological replication to strengthen statistical robustness and broaden the generalizability of the findings reported here. Moreover, although the absence of negative controls may represent a methodological limitation, this condition is inherent to field‐based studies, particularly those involving low‐biomass samples such as milk. However, the consistency observed among biological samples and the coherence of microbial patterns detected across distinct matrices indicate that the results obtained reliably reflect the actual microbial composition of the animals and their environment. Thus, even considering this limitation, the data generated provide a valid and relevant basis for the preliminary understanding of microbial interactions in buffalo production systems.

The information obtained from studies regarding the microbiome of these animals is essential for a better understanding of the importance of buffalo and calf well‐being, as well as providing valuable insight into the development of management strategies and interventions that promote a healthy microbiome in these animals.

## 5 Conclusion

Although somatic cell counts in milk differed between heifers and cows, the bacterial composition of the milk showed similarities between animals of different ages, resulting in a clustering of profiles independent of age.

Initially, the samples showed an animal‐dependent clustering pattern, followed by matrix‐dependent clustering, preventing the observation of clustering between generations of buffaloes and calves from first or third lactation.

Although differences in bacterial composition were observed between the matrices, 15 families were shared among the three matrices studied, strengthening the idea of interaction between the bacterial communities of each.

This article not only contributes to the knowledge of buffalo milk microbiology but also opens new avenues for future research, aiming at the advancement of milk production and quality, as well as promoting animal health.

## Author Contributions

**Allana Lais Alves Lima:** supervision, resources, investigation, funding acquisition, writing – original draft, methodology, validation, writing – review and editing, formal analysis, conceptualization. **Damazio Campos de Souza:** methodology, data curation, visualization, writing – review and editing, writing – original draft. **Rosiane do Socorro dos Reis:** methodology, data curation, investigation. **Diego Assis das Graças:** funding acquisition, visualization, software, formal analysis, data curation, supervision, methodology, resources, writing – review and editing, conceptualization. **Savio Souza Costa:** methodology, software, data curation, validation, investigation, conceptualization, formal analysis, writing – original draft. **Guilherme Costa Baião:** methodology, formal analysis, software, data curation. **Rinaldo Batista Viana:** methodology, formal analysis, funding acquisition, resources, supervision, validation, conceptualization, data curation. **Joana Montezano Marques:** supervision, resources, investigation, funding acquisition, writing – original draft, methodology, validation, writing – review and editing, formal analysis, conceptualization. **Rennan Garcias Moreira:** software, formal analysis, methodology.

## Funding

This work was supported by Coordenação de Aperfeiçoamento de Pessoal de Nível Superior (88887.633228/2021‐00).

## Ethics Statement

This study was conducted in accordance with the Animal Ethics and Welfare Committee of the Federal Rural University of the Amazon (UFRA).

## Conflicts of Interest

The authors declare no conflicts of interest.

## Supporting information


**Table S1:** Sequencing metrics per sample and alpha diversity values of faecal and milk samples from first‐ and third‐lactation buffaloes and saliva from their respective calves.
**Figure S1:** Rarefaction curve of faecal and milk samples from buffaloes and saliva samples from their respective calves.
**Figure** S**2**. Pearson correlation analysis between milk somatic cell count and alpha diversity index values. Micrococcales, Propionibacteriales, Rhodobacterales and Rhizobiales.

**Figure S3:** Rarefaction curves based on observed species richness for different sample classes (faeces, milk, soil and swabs). Each line represents an individual sample within each group.

## Data Availability

The data is available in the Sequence Read Archive (SRA) database under the accession number PRJNA1111038. The activity involving access to Genetic Heritage, as described in this study, was duly registered in the SisGen system under the registration number A144BC3, in compliance with Brazilian Law No. 13,123/2015 and its regulations.
