# Supplementary material for: Age Influences the Bacterial Composition of Samples From Buffaloes in the Marajó Archipelago, Pará, Brazilian Amazon
Source: Environ Microbiol Rep. 2026 Apr 28;18(3):e70330. doi: 10.1111/1758-2229.70330 (PMC13124447; doi:10.1111/1758-2229.70330)
Supplement: Supplementary file 1 — Table S1: Sequencing metrics per sample and alpha diversity values of faecal and milk samples from first‐ and third‐lactation buffaloes and saliva from their respective calves. Figure S1: Rarefaction curve of faecal and milk samples from buffaloes and saliva samples from their respective calves. Figure S2. Pearson correlation analysis between milk somatic cell count and alpha diversity index values. Micrococcales, Propionibacteriales, Rhodobacterales and Rhizobiales. [file EMI4-18-e70330-s002.docx]

Supplementary Material

**Age influences the bacterial composition of samples from buffaloes in the Marajó Archipelago, Pará, Brazilian Amazon**

**Allana Lais Alves Lima^1^; Savio Souza Costa^2^; Rosiane do Socorro dos Reis^2^; Damazio Campos de Souza^3^; Guilherme Costa Baião^4^; Rennan Garcias Moreira^4^; Herve Louis Ghislain Rogez^2^; Diego Assis das Graças^2^; Rinaldo Batista Viana^1^; Joana Montezano Marques^2^**

^1^ Institute of Health and Animal Production, Federal Rural University of Amazon, Belém, Brazil
^2^ Institute of Biological Sciences, Federal University of Pará, Belém, Brazil
^3^ Augusto Motta University (UNISUAM), Rio de Janeiro, Brazil
^4^ Institute of Biological Sciences, Federal University of Minas Gerais, Belo Horizonte, Brazil

^5^ Center for Natural and Human Sciences, Federal University of ABC, São Carlos, São Paulo, Brazil

*Correspondence: Joana Montezano Marques

jomontezanomarques@gmail.com

**Supplementary table** **1**. Sequencing metrics per sample and alpha diversity values of fecal and milk samples from first- and third-lactation buffaloes and saliva from their respective calves

| **Sample ID** | **Type** | **Observed** | **Chao 1** | **Shannon** | **Simpson** |
| --- | --- | --- | --- | --- | --- |
| 023-F** | Feces | 80 | 105 | 4.35 | 0.984 |
| 023-M** | Milk | 29 | 41 | 3.43 | 0.958 |
| 023-S** | Spittle | 114 | 166 | 4.89 | 0.991 |
| 036-F* | Feces | 56 | 89 | 4.32 | 0.984 |
| 036-M* | Milk | 40 | 64 | 3.68 | 0.970 |
| 036-S* | Spittle | 107 | 176 | 4.94 | 0.991 |
| 057-F* | Feces | 51 | 75 | 4.18 | 0.982 |
| 057-M* | Milk | 43 | 73 | 3.88 | 0.975 |
| 057-S* | Spittle | 96 | 126 | 4.59 | 0.987 |
| 427-F* | Feces | 56 | 83 | 4.27 | 0.984 |
| 427-M* | Milk | 37 | 41 | 3.48 | 0.965 |
| 427-S* | Spittle | 110 | 171 | 4.96 | 0.991 |
| 656-F** | Feces | 41 | 71 | 4.12 | 0.981 |
| 656-M** | Milk | 39 | 50 | 3.52 | 0.966 |
| 776-F** | Feces | 67 | 101 | 4.50 | 0.987 |
| 776-M** | Milk | 42 | 64 | 3.68 | 0.969 |
| 776-S** | Spittle | 105 | 173 | 4.97 | 0.991 |
|  |  |  |  |  |  |

Samples were identified by letter suffixes: M for milk, S for saliva, and F for feces; *Heifers; **Cows.


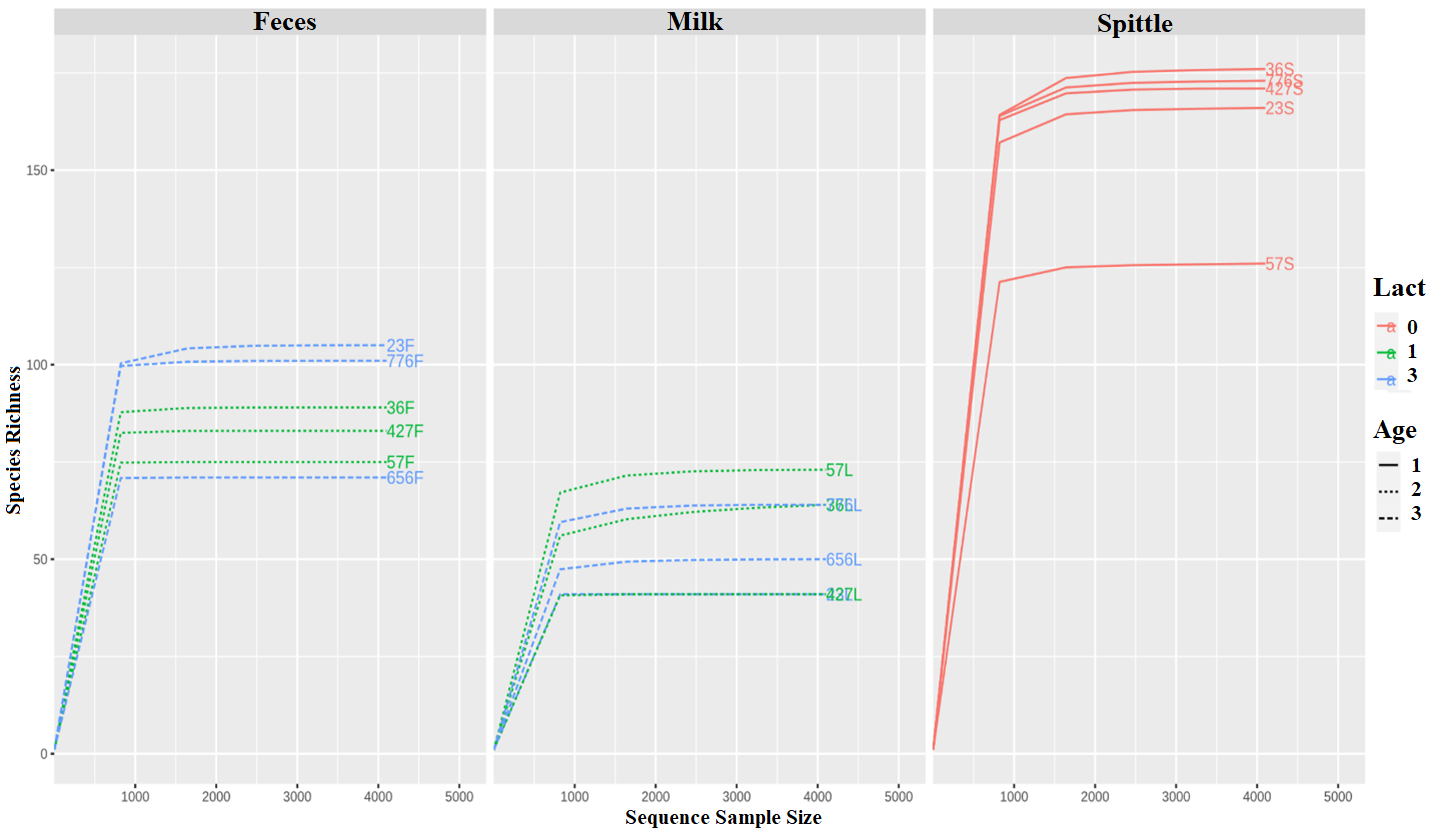


**Supplementary figure 1**. Rarefaction curve of fecal and milk samples from buffaloes and saliva samples from their respective calves.


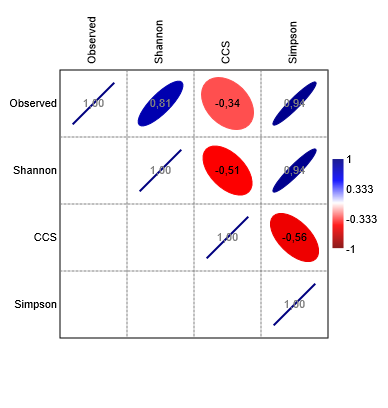


**Supplementary figure** **2.** Pearson correlation analysis between milk somatic cell count and alpha diversity index values. Micrococcales, Propionibacteriales, Rhodobacterales, and Rhizobiales.

BUB
