# Supplementary material for: Age Influences the Bacterial Composition of Samples From Buffaloes in the Marajó Archipelago, Pará, Brazilian Amazon
Source: Environ Microbiol Rep. 2026 Apr 28;18(3):e70330. doi: 10.1111/1758-2229.70330 (PMC13124447; doi:10.1111/1758-2229.70330)
Supplement: Supplementary file 2 — Figure S3: Rarefaction curves based on observed species richness for different sample classes (faeces, milk, soil and swabs). Each line represents an individual sample within each group. [file EMI4-18-e70330-s001.docx]

SUPPLEMNTARY MATERIAL


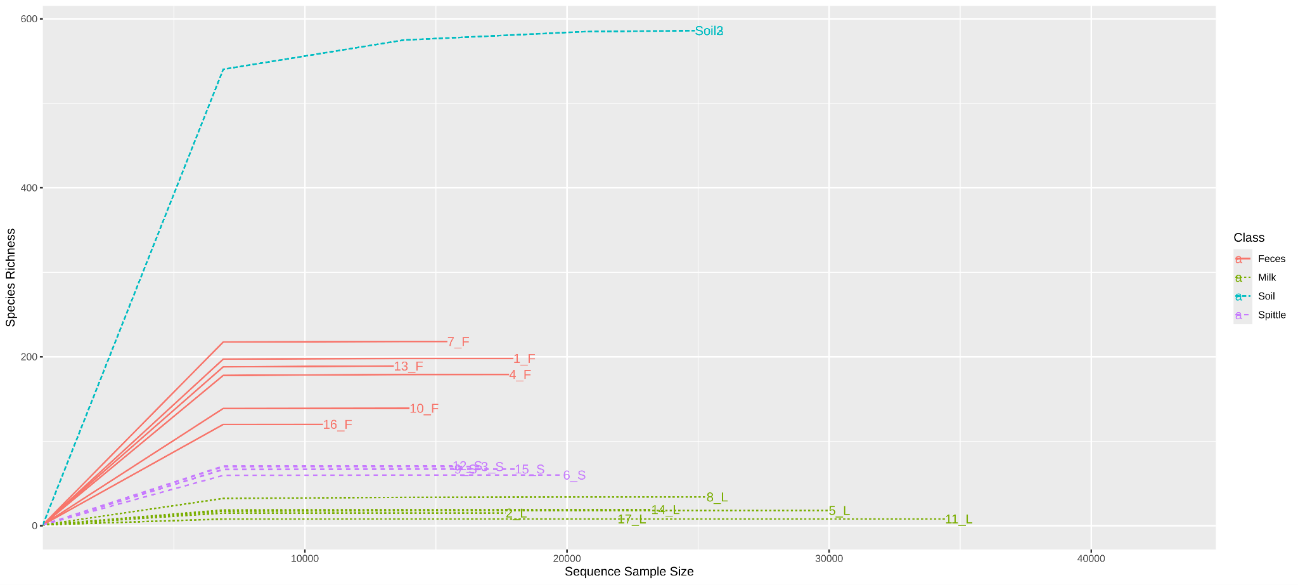


**Supplementary Figure S3.** Rarefaction curves based on observed species richness for different sample classes (feces, milk, soil, and swabs). Each line represents an individual sample within each group.

OS BIOMARCADORES
